# Bovine Staphylococcus aureus Superantigens Stimulate the Entire T Cell Repertoire of Cattle

**DOI:** 10.1128/IAI.00505-18

**Published:** 2018-10-25

**Authors:** Gillian J. Wilson, Stephen W. Tuffs, Bryan A. Wee, Keun Seok Seo, Nogi Park, Timothy Connelley, Caitriona M. Guinane, W. Ivan Morrison, J. Ross Fitzgerald

**Affiliations:** a The Roslin Institute, University of Edinburgh, Edinburgh, United Kingdom; b Institute of Infection, Immunity and Inflammation, University of Glasgow, Glasgow, United Kingdom; c Department of Basic Sciences, Mississippi State University, Mississippi State, Mississippi, USA; University of Illinois at Chicago

**Keywords:** Staphylococcus aureus, superantigen, T cell, cattle, mastitis

## Abstract

Superantigens (SAgs) represent a diverse family of bacterial toxins that induce Vβ-specific T cell proliferation associated with an array of important diseases in humans and animals, including mastitis of dairy cows. However, an understanding of the diversity and distribution of SAg genes among bovine Staphylococcus aureus strains and their role in the pathogenesis of mastitis is lacking. Population genomic analysis of 195 bovine S. aureus isolates representing 57 unique sequence types revealed that strains encode 2 to 13 distinct SAgs and that the majority of isolates contain 5 or more SAg genes. A genome-scale analysis of bovine reference strain RF122 revealed a complement of 11 predicted SAg genes, which were all expressed *in vitro*. Detection of specific antibodies in convalescent cows suggests expression of 7 of 11 SAgs during natural S. aureus infection. We determined the Vβ T cell activation profile for all functional SAgs encoded by RF122, revealing evidence for bovine host-specific activity among the recently identified RF122-encoded SAgs SElY and SElZ. Remarkably, we discovered that some strains have evolved the capacity to stimulate the entire T cell repertoire of cattle through an array of diverse SAgs, suggesting a key role in bovine immune evasion.

## Abstract (precis)

Superantigens (SAgs) represent a diverse family of bacterial toxins that induce Vβ-specific T cell proliferation associated with an array of important diseases in humans and animals, including mastitis of dairy cows. However, an understanding of the diversity and distribution of SAg genes among bovine Staphylococcus aureus strains and their role in the pathogenesis of mastitis is lacking.

## INTRODUCTION

Staphylococcus aureus produces a family of at least 26 distinct superantigens (SAgs), including the staphylococcal enterotoxins (SEs) SEA to -E, SEG to -J, and SER to -T; the staphylococcal enterotoxin-like toxins (SEls) SElK to -Q, -U, -V, and -X to -Z; and toxic shock syndrome toxin 1 (TSST-1) (1, 2). SAgs induce the Vβ-specific proliferation of T cells along with the release of proinflammatory cytokines, including interleukin-1 (IL-1), IL-2, IL-6, tumor necrosis factor alpha (TNF-α), and gamma interferon (IFN-γ), and the chemokines CCL2 and CCL3 (3, 4). The uncontrolled release of proinflammatory mediators can lead to rashes, fever, multiorgan damage, coma, and death from severe shock (1). The release of proinflammatory signals can impede the effectiveness of the immune response by creating a bias toward either the T_h_1 or T_h_17 response, disrupting the appropriate recruitment of effector cells (2). SAgs have been implicated in a wide range of human diseases, including staphylococcal food poisoning, endocarditis, necrotizing pneumonia, and severe toxic shock (1, 5–7). Taken together, the effects induced by SAgs are likely to cause a significant deficiency in the ability of the adaptive immune response to contribute effectively to clearance during S. aureus infection.

S. aureus is a common cause of bovine mastitis, an infection of the milk-secreting tissue of the udder, which represents a huge economic problem for the dairy industry worldwide (48, 49), establishing a typically chronic infection (8). The exact role of SAgs in this disease is currently unknown; however, it has been proposed that superantigenic activity may contribute to the persistence observed (9, 10). Although Vβ-specific activation of human T cells in response to staphylococcal SAgs has been well characterized (11–14), relatively little is known about Vβ-specific proliferation of bovine T cells. Previously, SEC and TSST-1 have been shown to induce Vβ-specific proliferation of bovine T cells (15–17). However, those studies were limited by the number of T cell receptor beta variable (TRBV) gene sequences available, with only 5 subfamilies, Vβ1, -2, -4, -13, and -28, included.

The bovine genome sequencing project and cDNA analyses led to the identification of the full complement of bovine Vβ subfamilies and almost the entire repertoire of bovine TRBV genes (18, 19). This facilitated the development of a quantitative real-time PCR (qRT-PCR) assay to study the bovine Vβ (bovVβ) response to stimulation with the core genome-encoded SAg SElX (14). S. aureus strain RF122 belongs to the common bovine-specific lineage sequence type 151 (ST151) and was the first animal-associated isolate to be fully sequenced (20). In this study, we have carried out a comprehensive, genome-wide analysis of the complement of SAgs encoded by this strain and determined the capacity of each toxin to activate bovVβ-specific T cells. We report host-specific functional activity for several SAgs and reveal the remarkable capacity of bovine S. aureus for activation of the full bovine T cell repertoire, suggesting a critical role in immune evasion. Importantly, we have also demonstrated that SAgs produced by S. aureus may play a role in the development of intramammary infection of dairy cows.

## RESULTS AND DISCUSSION

### Population genomic analysis indicates that bovine S. aureus strains contain 2 to 13 SAg genes.

**FIG 1:**
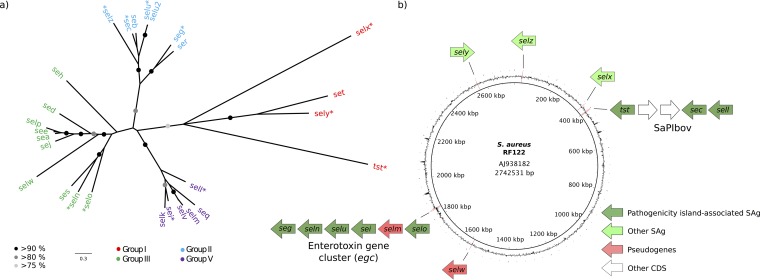
RF122 encodes SAgs from all four phylogenetic groups. (a) Maximum likelihood phylogenetic tree of 26 superantigen protein sequences showing clustering of SAgs into 4 general groups (1). Branches with more than 80% bootstrap support are marked with black or gray circles. SAgs present in the RF122 strain are indicated by asterisks. (b) Circular representation of the genome of reference strain RF122 showing the locations of SAg genes.

**FIG 2:**
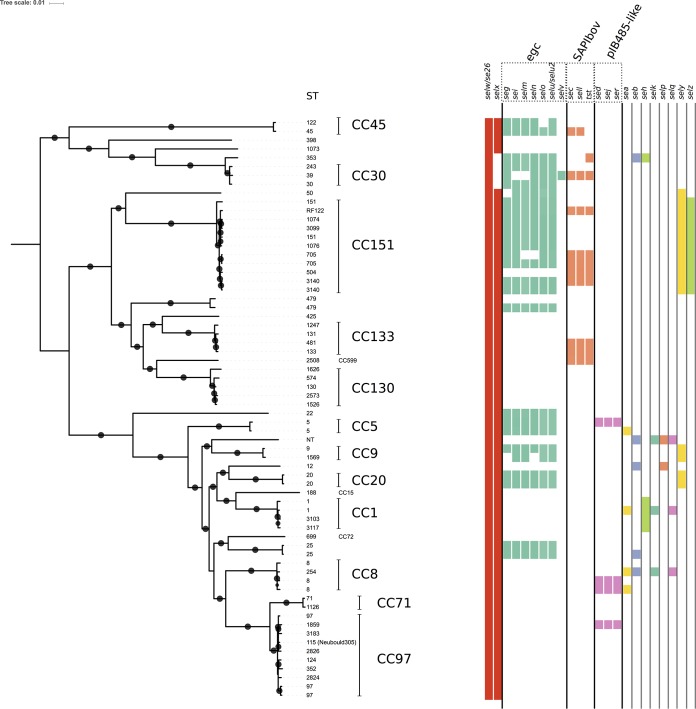
Bovine isolates of S. aureus typically contain 5 or more SAg genes. Distribution analysis of SAgs in bovine S. aureus isolates shows the repertoire of SAgs that are encoded. Phylogeny is based on a core genome alignment, and major clonal complexes are noted. Colored boxes indicate the presence of the SAg gene and are sorted according to association with mobile genetic elements.

We examined 195 bovine S. aureus genome sequences representing 57 unique sequence types (STs) for the presence of all 26 known members of the S. aureus SAg family (Fig. 1; see also Table S1 in the supplemental material). We employed a threshold of 90% sequence identity across the entire coding DNA sequence (CDS) to exclude cross-matches to other members of the same SAg group (Fig. 2; see also Table S1 in the supplemental material).

Consistent with data from previous studies, *selw* and *selx* were found in 100% (195/195) and 79% (150/195) of isolates analyzed, respectively. Previous studies identified *selw* to be inactivated in a large number of human S. aureus isolates examined, due to the lack of an ATG start codon (21, 22). However, the presence of alternative start codons (TTG) and a continuous full-length open reading frame and the high level of sequence conservation across bovine isolates (more than 94% nucleotide sequence identity) suggest that a high proportion of isolates have a functional *selw* gene. The *selx* gene was absent only in clonal complex 30 (CC30), consistent with data from previous reports (14). The *egc* cluster was present in 21 of the 57 unique STs analyzed and was highly prevalent within CC30, -151, and -45. The composition of *egc* varied, with six different gene arrangements characterized, but a gene complement of *seg*, *sei*, *selm*, *seln*, *selo*, and *selu-selu2* was the most common one observed. The bovine staphylococcal pathogenicity island (SaPI_bov_) was less prevalent than the *egc* cluster, found in 10 of the 57 STs analyzed, primarily in association with CC133 and CC151. The plasmid-borne SAg genes *sed*, *sej*, and *ser* were identified together in 4 strains, consistent with the presence of a pIB485-like plasmid as described previously in human strains (23). The *sely* and *selz* genes are distributed in a lineage-specific manner (CC151 and CC9 for *sely* and CC151 for *selz*), and the SAg genes *sea*, *seb*, *seh*, *selk*, *selp*, and *selq* were randomly distributed across the diversity of STs examined, consistent with horizontal gene transfer. The *set* and *ses* genes were not found in any S. aureus genomes examined, suggesting that they are not important in bovine pathogenesis.

All S. aureus strains examined contained at least 2 and up to 13 SAg genes. The majority of bovine STs analyzed (31/57) encode 5 or more SAgs, with CC151 isolates, such as RF122, generally encoding more SAgs (up to 13) than other bovine S. aureus strains. Fewer than half of the STs (*n* = 26) contained *selw* and *selx* only. An important example is the bovine reference strain Newbould 305, which has been the focus of a number of studies (24, 25), which encodes a functional copy of *selx* and a pseudogene of *selw* (25). The extensive variation in the SAg gene complements between Newbould 305 and RF122 may have a key impact on the relative pathogenesis of infections caused by these strains. Newbould 305 is associated with mild and generally subclinical infection, as opposed to RF122 and other CC151 isolates, which are associated with a more severe and clinical presentation of the disease (25, 26).

Analysis of the genome of bovine S. aureus isolate RF122 (GenBank accession number AJ938182) revealed a complement of 11 SAg genes and 2 SAg pseudogenes (Table 1, Fig. 1, and Table S1). Namely, RF122 contains the previously characterized SaPI_bov_ that contains *tst_bov_*, *sell_bov_*, *sec_bov_*, and the enterotoxin gene cluster (*egc*) in the genomic island vSaβ containing allelic variants of the SAg genes *seg*, *sei*, *selo*, *seln*, and *selu* and a pseudogene of *selm*. Spread out across other parts of the genome, RF122 also contains *selw* (pseudogene, SAB_1473c), *selx*, *sely*, and *selz* (Fig. 1). The SAg family was previously subdivided into phylogenetic groups I to V (group IV is composed entirely of streptococcal SAgs) (27, 28), and RF122 contains at least 2 genes from each of the 4 staphylococcal SAg subgroups (Fig. 1). Accordingly, RF122 was selected for genome-scale analysis of the expression and function of bovine S. aureus SAgs.

**TABLE 1:** SAgs encoded by S. aureus strain RF122

Gene	Toxin (abbreviation)	Size (kDa)^*a*^	Locus tag	Homology with characterized SAg gene (%) (characterized SAg gene)
*tst_bov_*	Toxic shock syndrome toxin 1 (TSST-1_bov_)	22	SAB_RS01910	98 (*tst*)
*sec_bov_*	Staphylococcal enterotoxin C-bovine (SEC_bov_)	27.6	SAB_RS01930	99 (*sec1*)
*sell_bov_*	Staphylococcal enterotoxin-like toxin L-bovine (SElL_bov_)	24.7	SAB_RS01935	99 (*sel1*)
*seg_bov_*	Staphylococcal enterotoxin G-bovine (SEG_bov_)	20.6	SAB1696c	77 (*seg1*)
*sei_bov_*	Staphylococcal enterotoxin I-bovine (SEI_bov_)	24.9	SAB_RS09045	97 (*sei1*)
*seln_bov_*	Staphylococcal enterotoxin-like toxin N-bovine (SElN_bov_)	26.1	SAB_RS09035	95 (*sen1*)
*selu_bov_*	Staphylococcal enterotoxin-like toxin U-bovine (SElU_bov_)	27.2	SAB_RS09040	97 (*selu1*)
*selm_bov_*	Staphylococcal enterotoxin-like toxin M-bovine (SElM_bov_)	NA	SAB1700c	87 (*selm1*)
*selo_bov_*	Staphylococcal enterotoxin-like toxin O-bovine (SElO_bov_)	27.1	SAB_RS09055	98 (*selo2*)
*selw*	Staphylococcal enterotoxin-like toxin W (SE26)	NA	SAB1473c	54 (*sea1*)
*selx_bov_*	Staphylococcal enterotoxin-like toxin X-bovine (SElX_bov_)	19.5	SAB_RS01710	45 (*tst*)
*sely_bov_*	Staphylococcal enterotoxin-like toxin Y-bovine (SElY)	22.5	SAB_RS13070	58 (*set*)
*selz_bov_*	Staphylococcal enterotoxin-like toxin Z-bovine (SElZ)	27.1	SAB_RS00140	57 (*seg1*)

a Predicted size of the mature protein based on the amino acid sequence. Pseudogenes are not included. NA, not applicable.

### Bovine SAg genes are expressed at different levels in a growth-phase-dependent manner *in vitro*.

**FIG 3:**
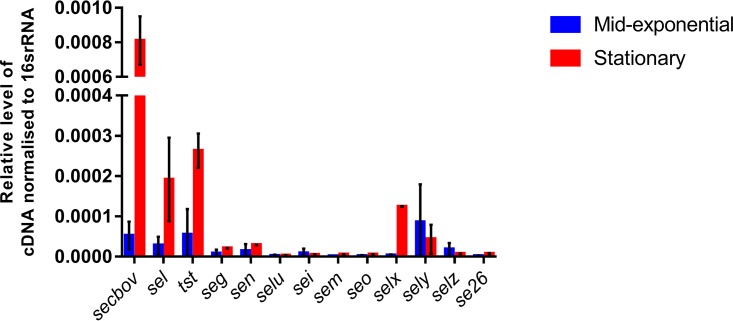
RF122 SAgs are expressed *in vitro* and exhibit growth-phase-dependent expression. Shown are transcription levels of RF122-borne SAg genes from exponential- and stationary-phase cultures, relative to 16S rRNA. Relative quantities of RF122 reverse-transcribed mRNA normalized to the internal control, 16S rRNA, were determined by qRT-PCR. Results shown are the means of data from triplicate experiments, and error bars indicate standard deviations (SD).

Relative transcriptional levels of RF122 SAg genes in the exponential and stationary phases of growth were determined by qRT-PCR. Transcription was detected for all 11 genes and 2 pseudogenes in both growth phases, with *sec_bov_* exhibiting the highest level of transcription and *selu* exhibiting the lowest (Fig. 3). Overall, SAg genes located on SaPI_bov_ were transcribed at higher levels than the *selx*, *sely*, *selz*, and *egc* genes and the SAg pseudogenes. The data indicate that SaPI_bov_ SAg genes and *selx* are upregulated in stationary phase, consistent with regulatory control by *agr*, whereas *sely* and *selz* are transcribed maximally in mid-exponential phase, suggesting *agr*-independent control. Of note, ST151 strains were previously demonstrated to have high levels of RNAIII transcription in comparison with other ruminant clones, which could provide an explanation for the high expression levels of some of these SAgs (26). In the present study, *egc* genes were transcribed at low levels, independent of the growth phase. This finding is consistent with those of Derzelle et al., who reported low *egc* transcript levels among 28 human strains (29). However, we cannot rule out the possibility that the *egc* genes are expressed at higher levels *in vivo*, as has been observed for the streptococcal SAgs SPEA and SPEC (30, 31). The differential regulation of SAg transcription *in vitro* suggests that SAgs are expressed at different stages of infection *in vivo*.

### S. aureus SAgs are expressed during bovine infection.

To determine if RF122-encoded SAgs are expressed during bovine infection, we produced recombinant proteins for each of the encoded SAgs and carried out Western immunoblot analysis with convalescent-phase sera from cows (Table 2). A serum sample obtained from a cow without a history of S. aureus mastitis did not contain antibody reactive to any of the SAgs tested and was used as a negative control (Table 2). IgG antibodies specific for 8 of the 11 SAgs were detected in at least 1 of the 4 bovine serum samples tested, whereas recombinant SEI_bov_ (rSEI_bov_), rSEG_bov_, and rSElO_bov_ were not reactive with any of the samples tested (Table 2). In a previous study by Wilson et al., rSElX_bov_ was demonstrated to be reactive with all bovine serum samples tested (14). Most human adults have antibodies specific for an array of S. aureus SAgs, including SEA, SEB, SEC, SED, SEE, SElX, and TSST-1, as a result of exposure during colonization or infection (14, 32, 33). The present study corroborates previous observations which showed that despite the relatively high prevalence of the *egc* cluster in clinical isolates of S. aureus, neutralizing antibodies are rare (34).

**TABLE 2:** Immunogenicity of recombinant SAg proteins from RF122 with sera from cows and humans with S. aureus infections

Serum sample^*b*^	Reactivity^*a*^										
	SElZ	SElY	SEG	SEI	SElO	SElU	SElN	SEC	SElL	TSST-1	SElX^*c*^
Human											
IE19	+	+	−	−	−	−	−	+	−	+	+
IE37	+	+	−	+	−	−	−	+	+	+	+
IE41	−	−	−	−	−	−	−	+	−	+	+
IE51	−	−	−	−	−	−	−	+	−	+	+
IE54	−	−	−	−	−	−	−	−	−	+	+
Bovine											
2480	+	−	−	−	−	−	−	−	−	−	+
2487	+	+	−	−	−	−	−	−	−	+	+
2521	+	+	−	−	−	+	−	+	+	+	+
4227	+	−	−	−	−	+	−	+	+	+	+
2211	−	−	−	−	−	−	−	−	−	−	−

a + or − indicates whether or not the serum sample is reactive with the SAg protein, respectively.

b Human serum samples were obtained from infective endocarditis patients between 2006 and 2009 at the New Royal Infirmary of Edinburgh. Bovine samples obtained from bovine mastitis cases, and from an animal (cow 2211) without a history of S. aureus infection, were provided by C. Smyth, originally obtained from the Teagasc Dairy Production Centre in Moorepark, Fermoy, County Cork, Ireland.

c Data reported previously (14).

This suggests that either the *egc* SAgs are poorly expressed during infection or the host is unable to generate antibodies due to low T or B cell reactivity. Importantly, in this study, we have shown that SElY, SElZ, and, to a lesser extent, SElU and SEI are expressed by S. aureus in vivo. Antibodies against SElY and SElZ have been detected in at least one serum sample each of bovine and human origin, consistent with a role in pathogenesis in both host species.

Although our data suggest low levels of expression of some SAgs, it is feasible that they can contribute to S. aureus immune modulation. For example, we recently demonstrated that suboptimal stimulation of human T cells with a low concentration of SAg (1 ng/ml) induced CD8^+^ CD25^+^ FOXP3^+^ regulatory T cells that strongly suppress the activation of effector T cells (35). A similar phenomenon can be observed in the bovine system, as immunosuppressive CD4^+^ CD25^+^ FOXP3^+^ cells are activated with equivocally low concentrations of SAg (1 ng/ml) (36).

### RF122-encoded SAgs are mitogenic for bovine T cells.

In order to examine the mitogenicity of each of the 11 identified SAgs, we constructed a SAg-deficient mutant of S. aureus strain RF122 to facilitate plasmid-mediated expression of each SAg in isolation by its native S. aureus strain. S. aureus RF122-1, a TSST-1-deficient derivative of RF122, was constructed previously by allele replacement of the *tst* gene with a tetracycline resistance cassette (17). In turn, we sequentially deleted the *sec*, *sel*, *egc*, *selx*, *sely*, and *selz* genes by allele replacement (see Fig. S1 in the supplemental material), resulting in the sequential mutants RF122-2 to RF122-7 and the final SAg-deficient derivative RF122-8 (Table S3 and Fig. S1). Finally, to limit Hla-mediated toxicity for T cells, we constructed *hla* mutants in the parent strain RF122 and SAg-deficient derivatives, resulting in strains RF122t-α and RF122-8α, respectively (Table S3). The mutants were validated to rule out that spurious mutations accrued during *in vitro* passage that impact secreted virulence proteins (Fig. S2). Analysis of the mitogenicity of stationary-phase and mid-exponential-phase culture supernatants of RF122 and RF122-8 confirmed the loss of all detectable mitogenic activity (Fig. S2).

**FIG 4:**
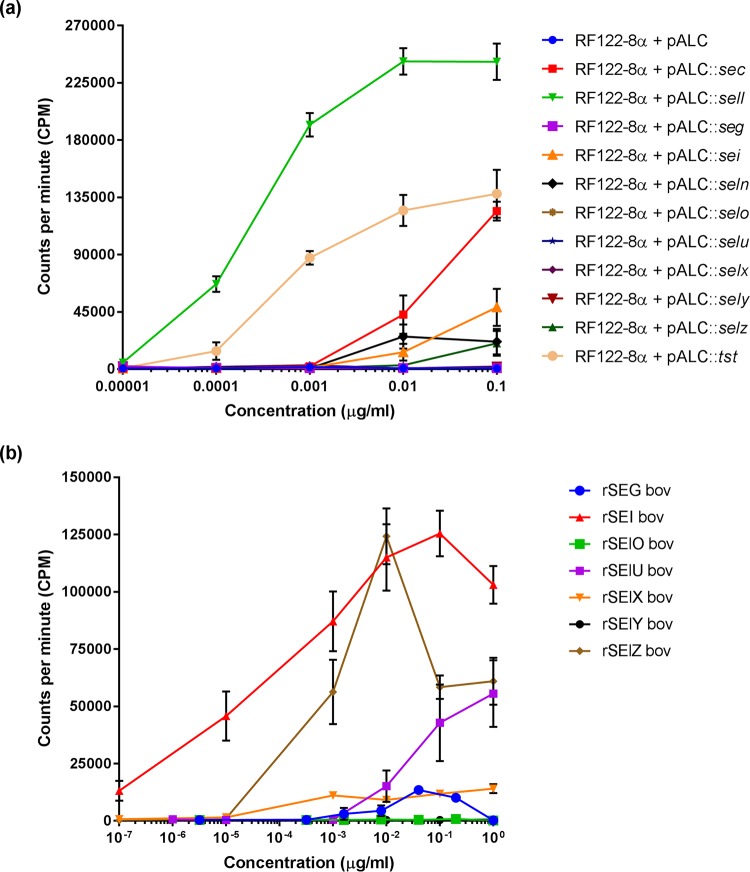
Proliferation of bovine T cell populations in response to stimulation with RF122-encoded SAgs. Shown are data for PBMC proliferation after 4 days of exposure to RF122-8 supernatants containing SAgs (a) and recombinant SAg proteins (b), as indicated by the incorporation of [^3^H]thymidine. Results shown are the means of data from at least triplicate measurements from 2 animals ± standard errors of the means (SEM).

The superantigenic activities of RF122-encoded SEC_bov_, TSST-1_bov_ and SElX_bov_ were described previously (14–17). In order to examine the mitogenic potential of all SAgs encoded by RF122 expressed in a native S. aureus background, SAg genes were cloned into the inducible expression plasmid pALC2073. This allowed controlled expression in the SAg-deficient strain RF122-8α, facilitating analysis of the effects of individual SAgs produced in their native strain context on bovine T cells *in vitro*. Proteins of the predicted molecular weights were detected in supernatants of induced RF122-8α cultures for each SAg plasmid construct, with the exceptions of SEG_bov_, SElN_bov_, SEI_bov_, and SElO_bov_. (Fig. S3). To examine the mitogenicity of RF122-encoded SAgs for bovine T cells, culture supernatants of RF122-8α containing pALC2073::SAg constructs and recombinant SAg proteins were used to stimulate bovine peripheral blood mononuclear cells (PBMCs), and proliferation was measured by using a thymidine incorporation assay (Fig. 4). Mitogenic activity for bovine T cells was detected for 7 of the 11 SAgs expressed in the SAg-free strain RF122-8α, including TSST-1, SEC_bov_, SEL_bov_, SEI_bov_, SElN_bov_, SElX, and SElZ_bov_, at total protein concentrations ranging from 10 pg/μl to 10 ng/ml, but there was no detectable mitogenic activity for SElO_bov_, SEG_bov_, SElU_bov_, and SElY (Fig. 4a). However, recombinant proteins rSEG_bov_, rSElU_bov_, and rSElY expressed in Escherichia coli could stimulate T cell proliferation at higher concentrations (Fig. 4b). Accordingly, of the 11 SAgs encoded by RF122, only SElO_bov_ did not exhibit any capacity for stimulation of bovine T cells. Taken together, these data indicate that RF122 encodes an array of SAgs that are potent bovine T cell mitogens.

### RF122-encoded SAgs have the capacity to stimulate the entire bovine Vβ repertoire.

**FIG 5:**
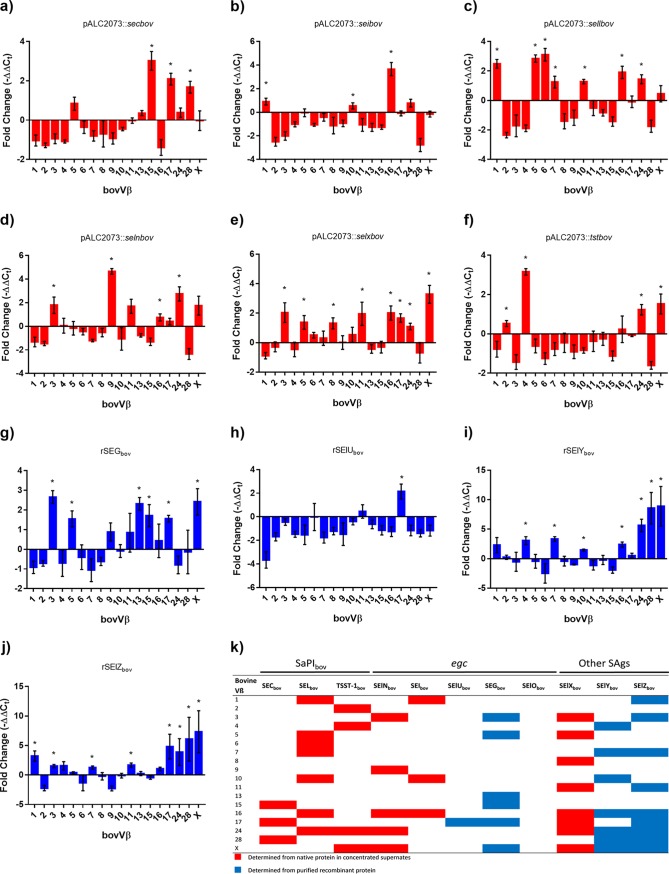
RF122-encoded SAgs are able to stimulate all Vβ subsets of the bovine T cell population. (a to j) Relative fold changes in bovine Vβ expression after stimulation with RF122 SAgs. Bovine Vβ subfamilies were named according to the classification described previously by Arden et al. (47). The bovine TRBV genes analyzed are functional genes tested previously (14). Bovine T cells were stimulated with supernatants from induced RF122-8α cells containing pALC2073::SAg constructs (a to f) or purified recombinant proteins (g to j). Results are given as mean fold changes in expression ± SEM from 6 measurements, 3 each from two animals. * indicates expansion of a subfamily based on a significant increase from the baseline (*P* < 0.05). (k) Expansion profiles of all 11 SAgs from RF122.

**FIG 6:**
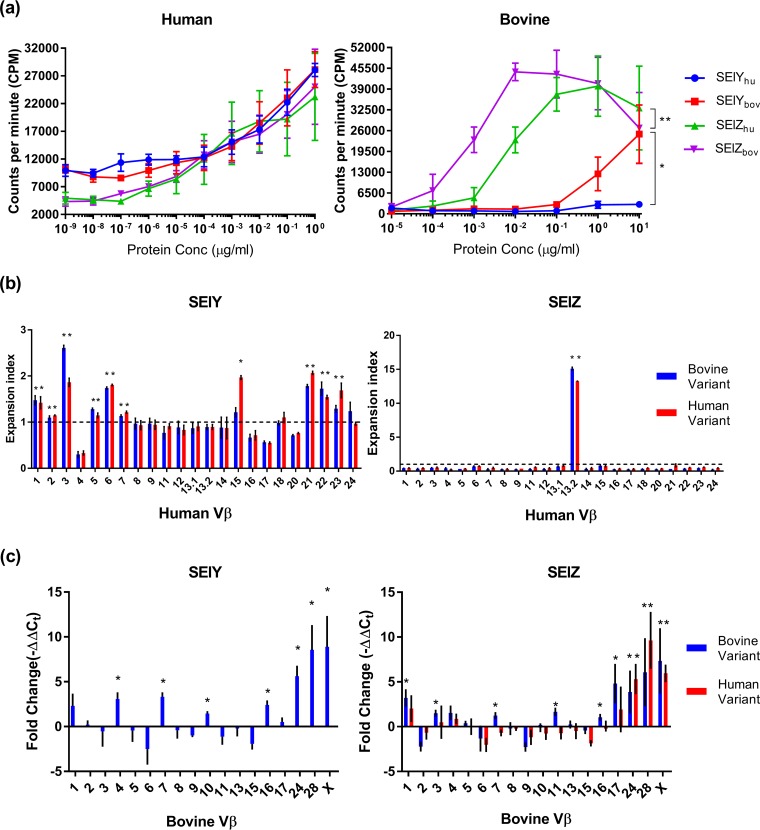
SAgs exhibit host-dependent functional activity. (a) PBMC proliferation after 4 days of exposure to bovine and human alleles of SElY and SElZ as indicated by the incorporation of [^3^H]thymidine. Results shown are the means of data from at least triplicate measurements from 3 donors ± SEM. Differences between proliferation induced by human and bovine variants of these SAgs were assessed by using two-way analysis of variance (ANOVA) with a Holm-Sidak multiple-comparison test, and asterisks denote curves that are significantly different (*, *P* < 0.05; **, *P* < 0.01). (b) Expansion index of Vβ human CD3^+^ cells after stimulation with human and bovine alleles of SElY and SElZ. The expansion index was determined from the means of three measurements from 2 donors ± SEM. * indicates expansion of a subfamily based on a significant increase from the baseline (*P* < 0.05) and an expansion index of >1. (c) Relative fold changes in bovine Vβ expression after stimulation with human and bovine alleles of SElY and SElZ. Results are given as mean fold changes in expression ± SEM from 9 measurements, 3 each from three animals. * indicates expansion of a subfamily based on a significant increase from the baseline (*P* < 0.05).

Most previous studies of the bovine Vβ-dependent T cell activation capacity of staphylococcal SAgs have been limited by the number of identified bovine Vβ subfamilies (15, 17). Recently, we developed a novel qRT-PCR assay that is representative of the full complement of bovine Vβ subfamilies (14). Supernatants from tetracycline-induced cultures of RF122-8α containing pALC2073::SAg constructs were used to stimulate bovine T cells. If the supernatant was unable to induce proliferation at a total protein concentration of 0.01 μg/ml, purified recombinant protein was used as an alternative to determine the bovVβ profile (Fig. 5). Accordingly, in the present study, we were able to comprehensively evaluate the responses of 18 bovVβ subfamilies to stimulation with all RF122-encoded SAgs by qRT-PCR (Table 3 and Fig. 5). In order to examine the host specificity of bovine SAgs, we also examined the capacities of the recently characterized SAgs SElY and SElZ to stimulate Vβ-dependent activation of human T cells (Fig. 6 and Table 3). We found that all SAgs encoded by RF122, with the exception of SElO_bov_, induced Vβ-specific stimulation of bovine T cells (Fig. 5), with a unique bovVβ activation profile similar to human Vβ (humVβ) T cell activation profiles (37). Of note, the data indicate that each of the 18 bovVβ subfamilies tested are activated by at least one RF122-encoded SAg, such that RF122 has the potential to stimulate the entire bovVβ repertoire (Fig. 5). Remarkably, the 3 SAgs encoded by SaPI_bov_ alone activate 13 of 18 bovVβ subfamilies, highlighting the potential importance of SaPI_bov_ in bovine immune evasion. In comparison, despite being twice in number, the *egc* SAgs activate only 11 of 18 subfamilies. Extensive duplication within the bovVβ repertoire has resulted in 9 multimember subgroups, the largest of which, bovVβ1, -10, and -13, contain 23, 9, and 20 functional TRBV genes, respectively (18, 19). Each of the SaPI_bov_-encoded SAgs, SEC_bov_, SElL_bov_, and TSST-1_bov_ and the *egc*-encoded SAg SEI_bov_ can activate at least one of these large subfamilies each (Fig. 5). SElL_bov_ activates both bovVβ1 and -10, which is consistent with the large proportion of T cells that are induced in response to stimulation with this SAg (Fig. 4 and 5). It was shown previously that all humVβ subfamilies (with the exception of humVβ4 and -11) are activated by at least one SAg (2). Our data also indicate that some bovVβ subfamilies can be activated by multiple SAgs, for example, Vβ16 and -X are activated by 6 RF122-encoded SAgs, and Vβ24 and -17 are activated by 5 of them. This apparent functional redundancy implies that the activation of these Vβ subfamilies is of critical importance in S. aureus infection. A similar redundancy has been observed in the humVβ response to SAgs, with Vβ1, -3, -5, -6, -9, -12, -18, and -21 being targeted by at least 5 or more different SAgs (2).

**TABLE 3:** Activation of Vβ subfamilies in response to RF122-encoded SAgs

SAg	Bovine Vβ subfamily(ies) activated^*a*^	Human Vβ subfamily(ies) activated^*a*^^,^^*b*^
SEC_bov_	**15**, **17**, 28	12, **13**, 14, **15**, **17**, 20
SEI_bov_	**1**, 10, 16	**1**, 5, 6, 23
SElL_bov_	**1**, **5**, 6, **7**, 10, **16**, 24	**1**, **5**, **7**, **16**, 22, 23
SElN_bov_	3, **9**, 16, 24, X	7, 8, **9**, 17
SElX_bov_	3, 5, 8, 11, 16, 17, 24, X	1, 6, 18, 21
TSST-1_bov_	**2**, 4, 24, X	**2**
SEG_bov_	3, 5, 13, 15, 17, X	3, 12, 13, 14, 15
SElU_bov_	17	13, 14
SElY_bov_	4, **7**, 10, 16, **24**, 28, X	1, 2, 3, 5, 6, **7**, 15, 21, 22, 23, **24**
SElZ_bov_	1, 3, 7, 11, 16, 17, 24, 28, X	13.2
SElO_bov_	NA	5, 7

a Vβ subfamilies were named according to the classification described previously by Arden et al. (47). Bovine and human Vβ subfamilies activated in response to the same SAg are highlighted in boldface type. NA, not applicable.

b Human Vβ activation data were compiled previously or in this study for SElY and SElZ (11, 12, 14, 15).

### Evidence for host adaptation by bovine S. aureus SAgs.

For the recently characterized SAgs SElY and SElZ, we examined the Vβ-dependent activation of human and bovine T cells. We utilized protein variants for both SElY and SElZ derived from human and bovine isolates to investigate the possibility of host adaptation. Both human and bovine variants of SElY and SElZ induced similar levels of expansion of human T cells (Fig. 6a). SElY induced the expansion of a broad number of human Vβ subfamilies (Fig. 6b), while SElZ induced the expansion of a single human Vβ subfamily (subfamily 13.2). In contrast to the human Vβ expansion profile, both human and bovine variants of SElY and SElZ activated different bovVβ subfamilies. SElZ_bov_ activated bovVβ subfamilies 1, 3, 7, 11, 16, 17, 24, 28, and X, while human SElZ (SElZ_hum_) activated bovVβ subfamilies 24, 28, and X (Fig. 6c). It is also noteworthy that SElZ_bov_ exhibited a 10-fold-higher potency than SElZ_hum_ for stimulating bovine T cell proliferation (Fig. 6a). This could be explained by the activation of a broader number of bovVβ subfamilies by SElZ_bov_ than by the human variant (Fig. 6c). Strikingly, SElY_bov_ induced the expansion of a broad array of bovVβ subfamilies, while SElY_hum_ was unable to induce the activation of bovine T cells (Fig. 6a and c). Combined, these results suggest adaptive evolution of SElY and SElZ to the bovine host.

Analysis of the protein variants SElY and SElZ revealed a number of unique residues that may be responsible for the difference in phenotypes observed between the human and bovine variants (see Fig. S4 in the supplemental material). For SElY, three positions varied between the bovine allele from RF122 (ST151) and the human allele from MSA2020 (ST121) (E19G, T67A, and I183V). In particular, the glutamic acid residue at position 19 was identified in the SElY allele of ST151 and other cattle isolates (ST3140, -504, -706, and -3099) but not in any of the SElY variants of human origin. For SElZ, four positions varied between the bovine allele from RF122 (ST151) and the human allele from MSA1695 (P6L, N55S, D75N, and G106A). Of note, the glycine residue at position 106 of RF122 SElZ was found in all but one of the bovine SElZ variants analyzed and was absent among the majority of human variants (6/8).

Some of the differences between human and bovine Vβ activation profiles are due to the absence of an orthologous subgroup, such as the activation of humVβ12, -14, -20, -22, and -23 (absent in bovine) and bovVβ10, -28, and -X (absent in human) (18). However, there are cases where Vβ subfamilies from one host are activated but the orthologous subgroup from the other is not (Table 3). For example, SElL_bov_ activates bovVβ6 and -24 but not humVβ6 and -24, TSST-1_bov_ activates only bovine Vβ4 and -24, SEI_bov_ activates bovVβ16 and humVβ5 and -6 but not the equivocal variants in the opposite species, and SElN_bov_ activates bovVβ3, -16, and -24 and humVβ7 and -8 but not the equivalent human or bovine subgroups. It is important to note that with the exception of SEC_bov_ and SElX_bov_, the human Vβ profiles described here were determined in previous reports in response to stimulation with SAgs derived from human S. aureus strains (11, 12). It is feasible that distinct human Vβ profiles could be stimulated by bovine SAg variants. Our analysis of SElY and SElZ and a previous analysis of SElX (14) support the notion that allelic variants of SAgs made by S. aureus from different host species have evolved to preferentially activate the Vβ repertoire of the strains of the target host. Together, these data indicate that some SAgs encoded by bovine S. aureus have undergone host adaptation associated with broader stimulation of Vβ subfamilies and increased potency of bovine T cell activation. Furthermore, we report that SElY and SElZ are classical SAgs in that they have unique Vβ activation profiles with the capacity to mediate immune modulation in both humans and cattle.

### Preliminary examination of the role of SAgs in the pathogenesis of bovine mastitis.

**FIG 7:**
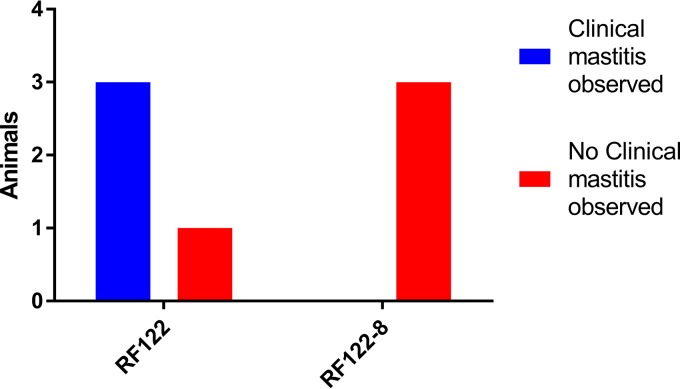
SAgs promote clinical bovine mastitis. Shown is the number of animals infected with RF122 or RF122-8 that exhibited evidence of clinical mastitis at any point during the 21 days of the trail. Clinical mastitis in this experiment was defined as observable inflammation in any of the four quarters of the cow's udder during the study.

The functional analysis of bovine SAgs made by a single strain in the present study suggests a profound role in host-pathogen interactions and pathogenesis. In order to examine the role of SAgs in S. aureus bovine mastitis, preliminary experimental infections of bovine mammary glands were carried out using RF122 and RF122-8 over a course of 21 days. Seven healthy dairy cows in their 1st to 4th lactation were enrolled in two groups of 4 and 3 cows and challenged with wild-type (WT) RF122t and the SAg-deficient strain RF122-8, respectively. There were no differences observed between the groups in terms of somatic cell counts, milk yields, and core body temperatures (see Fig. S5 in the supplemental material). S. aureus was isolated from the mammary glands of all animals during the trial; taken together with the milk quality and somatic cell counts, these data indicate that SAgs are not required to establish subclinical mastitis. The group infected with wild-type RF122 exhibited clinical mastitis at least once, in three out of the four animals infected during the course of the study (Fig. 7). In contrast, clinical mastitis was not observed in the animals infected with the SAg-deficient mutant. Although the study was not powered for statistical significance, the data are suggestive of a role for bovine SAgs in the development of staphylococcal clinical mastitis. Further experimentation would be required to confirm this preliminary observation.

We speculate that SAgs may contribute to pathogenesis through the expression of some SAgs, such as SEC1 and TSST-1, at high concentrations to promote the release of proinflammatory cytokines, which in turn induce tissue damage, inflammation, and clinical pathology. Furthermore, some SAgs, such as *egc*-encoded SAgs, expressed at low concentrations may induce immunosuppressive regulatory T cells to promote colonization of the host.

### Concluding comments.

In conclusion, the role of SAgs during pathogenesis is very complex. The array of identifiable staphylococcal SAgs is expanding and has been expedited with advances in genomic analyses. The extensive diversity is potentially driven by the need to activate a large number of T cells and bind to major histocompatibility complex (MHC) class II molecules in multiple ways, contributing to immune evasion. Our findings contribute to the understanding of staphylococcal SAg diversity and provide a comprehensive analysis of the bovine T cell response to SAgs. In addition, we report examples of toxins that contribute to the capacity of S. aureus to adapt to different host species.

## MATERIALS AND METHODS

### Ethics statement.

All *in vivo* work was done after local ethical review, under the oversight of the Kalamazoo IACUC, and in accordance with local, state, and federal animal welfare regulations. Bovine venous blood was taken under the authority of a UK Home Office project license (PPL 604394) within the terms and conditions of the regulations of the UK Home Office Animals (Scientific Procedures) Act of 1986 and the code of practice for the housing and care of animals bred, supplied, or used for scientific purposes. Human venous blood was taken from healthy donors in accordance with a human subject protocol approved by the National Research Ethics Service (NRES) Committee of South East Scotland under research ethics committee reference number 11/AL/0168. Volunteers were recruited by a passive advertising campaign within The Roslin Institute (University of Edinburgh), and written consent was given by each volunteer before each sample was taken.

### Bacterial culture conditions.

S. aureus strains were grown in tryptone soya broth (TSB) or brain heart infusion (BHI) broth (Oxoid, UK) shaken at 200 rpm or on tryptone soya agar (TSA) (Oxoid, UK) at 37°C for 16 h unless otherwise stated. E. coli strains were grown in Luria-Bertani (LB) broth (Melford Laboratories, UK) shaken at 200 rpm or on LB agar (Melford Laboratories, UK) at 37°C for 16 h unless otherwise stated. Media were supplemented where appropriate with 150 μg/ml X-gal (5-bromo-4-chloro-3-indolyl-β-d-galactopyranoside), 50 μg/ml ampicillin, and 10 μg/ml erythromycin or chloramphenicol (Sigma-Aldrich, Dorset, UK). For growth curve analysis of S. aureus, strains were cultured overnight in 5 ml BHI broth (Oxoid Ltd., Basingstoke, UK) in triplicate. After 12 h, strains were subcultured at a dilution of 1:100 into 30 ml fresh BHI broth in 250-ml Erlenmeyer flasks and placed in a shaking incubator at 37°C at 200 rpm. Absorbance readings were measured at 600 nm (optical density at 600 nm [OD_600_]) using a spectrophotometer (Cecil Aurius CE2021; Thistle Scientific Ltd., Glasgow, UK) over a period of 12 h, and a growth curve was determined.

### Sequence analysis of staphylococcal SAg genes.

The sequences of characterized staphylococcal SAg genes were obtained from the NCBI GenBank database (see Table S1 in the supplemental material). SAg homologs were identified in publicly available whole-genome sequences of bovine and representative human S. aureus genomes using BLASTn with a minimum alignment of 90% nucleotide identity averaged across the entire gene sequence using the Blastable script (https://github.com/bawee/blastable). Representative genomes with unique sequence types and SAg contents were selected, and a core genome alignment was built using Parsnp (38). The association between SAg content and phylogeny was visualized using iTol (39). Nucleotide sequences corresponding to each reference SAg were aligned at the codon level using translatorx and mafft (40, 41). A maximum likelihood tree was constructed from the translated amino acid alignment using RAxML (v8.2.10) with the following settings: —m PROTCATAUTO —f a —N 1000 —x 123 —p 123 (42). BRIG (43) was used to construct the circular genome representation and GC content plot with the S. aureus RF122 genome sequence (GenBank accession number AJ938182) as a reference.

### Transcriptional analysis of SAg genes.

Total RNA was extracted from S. aureus strain RF122 exponential-phase (OD_600_ = 0.6) and stationary-phase (12-h) cultures using the RNeasy miniprep kit (Qiagen, UK) according to the manufacturer's instructions except for an added lysis step with resuspension of the bacterial pellet in Tris-EDTA (TE) buffer with 100 μg/ml lysostaphin and incubation at 37°C for 20 min. RNA was treated with Turbo DNase (Thermo Fisher, UK). A total of 0.5 μg mRNA was analyzed for gene transcription using the same protocol as the one outlined by Wilson et al. (14). SAg primers are listed in Table S2 in the supplemental material.

### Allelic replacement of SAg genes.

Gene deletion constructs of SAg genes in RF122 were performed by using constructs prepared in plasmid pMAD (44) (see Table S3 in the supplemental material). Plasmid construction and allelic replacement were performed as described previously (14, 44). The resulting mutant strain, which had lost the gene of interest (GOI), was analyzed by PCR for no amplification with primers within the deleted region or with pMAD MCS primers (see Table S2 in the supplemental material). The mutant strains were also sequenced by using primers upstream (E) and downstream (Z) of the GOI to confirm the predicted deletion event. Sequencing reactions were carried out by Edinburgh Genomics (King's Buildings, University of Edinburgh, UK). To investigate the possibility that deletion of the genes could have pleiotropic effects, the phenotypes of WT and mutant strains were compared. First, a growth curve was determined for RF122, RF122t, and RF122-8, grown in a BHI liquid culture for 10 h at 37°C, which revealed that growth rates and yields were similar for each strain (Fig. S2). In addition, the hemolysis of rabbit erythrocytes incubated with culture supernatants of RF122 and SAg-deficient derivative strains was investigated. In each case, the hemolytic titer of RF122 and SAg-deficient derivatives was 1,022, indicating that the deletion of SAg genes had no effect on hemolytic activity and that the *agr* locus was functional (Fig. S2). Deletion of the *hla* gene in RF122 resulted in a reduction in the hemolytic titer, indicating that these strains are less toxic than the wild type. Analysis of the profiles of secreted and cell wall-associated (CWA) proteins of WT and mutant strains revealed no unexpected differences (Fig. S2).

### Analysis of S. aureus secreted and CWA proteins.

Secreted and CWA proteins were extracted from S. aureus mid-exponential-phase (OD_600_ = 0.6) and stationary-phase (12-h) cultures grown in BHI medium. Cells were centrifuged at 4,000 × *g*, and supernatant fractions containing secreted proteins were removed and concentrated with Amicon Ultra-15 centrifugal filter units with a 10-kDa molecular weight cutoff (MWCO) according to the manufacturer's instructions (Merck Millipore, UK). To extract CWA proteins, pelleted cells were washed with 1 ml phosphate-buffered saline (PBS) (Oxoid, Cambridge, UK), resuspended in 1 ml lysis buffer (50 mM Tris HCl, 20 mM MgCl_2_, and 30% raffinose [Fluka, UK], adjusted to pH 7.5) containing 200 μg/ml lysostaphin (AMBI Products LLC, NY, USA) and protease inhibitors (Roche, UK), and incubated at 37°C for 20 min. Samples were centrifuged at 6,000 × *g* for 20 min, and CWA proteins were recovered from the supernatant fraction. Protein preparations were separated on 10% SDS-PAGE gels, stained overnight at room temperature with Coomassie blue (Severn Biotech), or transferred to nitrocellulose membranes (Amersham Hybond ECL; GE Healthcare, Slough, UK) for Western blot analysis. The membrane was incubated with primary antibody for 1 h with a 1:2,500 dilution of anti-SEC (Santa Cruz Biotechnology, Heidelberg, Germany) or for 2 h with a 1:2,000 dilution of rat antiserum specific for rTSST-1, rSElL, or rSElX_bov_. The membrane was incubated with secondary antibody for 1 h at a dilution of 1:2,500 (rabbit anti-mouse IgG; Zymed, Invitrogen, UK) or 1:1,500 (goat polyclonal antibody to rat IgG-horseradish peroxidase [HRP]; Abcam, Cambridge, UK) and visualized by enhanced chemiluminescence (ECL).

### Cloning of SAg genes into pALC2073.

5′ oligonucleotides to amplify RF122-borne SAg genes for cloning into the expression plasmid pALC2073 were designed to prime upstream of the predicted ribosome binding site (RBS) with a KpnI site incorporated to facilitate cloning (see Table S2 in the supplemental material). The 3′ primer was designed to include the stop codon of the gene with a SacI site incorporated (Table S2). PCRs were carried out with 10 ng RF122 genomic DNA (gDNA) and 100 nmol forward and reverse primers, as listed in Table S2 in the supplemental material, using 1 U Vent polymerase (New England BioLabs, Herts, UK) according to the manufacturer's instructions. PCR products were cloned into the Strataclone pSC-B plasmid (Agilent, Cheshire, UK), and inserts were released by digestion with SacI and KpnI for 3 h at 37°C, purified by gel extraction, ligated with digested pALC2073 plasmid DNA using T4 DNA ligase, and transformed into E. coli DH5α cells. The resulting pALC2073::SAg plasmids were isolated from DH5α cells and transformed by electroporation into an intermediate electrocompetent strain of S. aureus, RN4220. Subsequently, the plasmids were reisolated and transformed into the SAg-deficient strain RF122-8. S. aureus strains were made competent as described previously (14). RF122-8 strains containing each of the pALC2073::SAg constructs were induced with a subinhibitory concentration of tetracycline (50 ng/ml) (Sigma-Aldrich, Dorset, UK) when cultures reached mid-exponential phase and grown for a further 4 h.

### Recombinant expression of SAg genes.

5′ primers for cloning into the pET15b (Merck Millipore, UK) or pQE30-Xa (Qiagen, UK) plasmid were designed to anneal immediately after the signal peptide coding region, as predicted by the Signal P 3.0 server (http://www.cbs.dtu.dk/services/SignalP/), and 3′ primers were designed to include the stop codon of the gene (see Table S2 in the supplemental material). The cloning procedure was performed as outlined above for pALC2073, and ligated constructs were transformed into E. coli DH5α or XL1-Blue (for pQE30-Xa constructs) cells. pET constructs were isolated from DH5α cells using the QIAprep spin miniprep kit (Qiagen, UK) and transformed into E. coli BL21(DE3) cells. BL21 or XL1-Blue cells containing expression constructs were cultured in Luria broth containing 50 μg/ml ampicillin (Sigma-Aldrich, Dorset, UK) and induced in the mid-exponential phase of growth (OD_600_ = 0.6) with 1 mM isopropyl-β-d-1-thiogalactopyranoside (IPTG) (ForMedium Ltd., Norfolk, UK) for 4 h. Cells were recovered by centrifugation at 8,000 × *g* and disrupted by using a French press, and His-tagged recombinant proteins were purified by affinity chromatography on a Ni-nitrilotriacetic acid (NTA) nickel affinity column (GE Healthcare, UK). Proteins were dialyzed by using Spectra/Por Float-A-Lyzer tubing with an 8,000 to 10,000 MWCO (Spectrum Laboratories, CA, USA).

### Immunoblot analysis of convalescent-phase bovine serum.

SDS-PAGE and Western blotting were carried out on SAgs overexpressed in E. coli. The nitrocellulose membrane (Amersham Hybond ECL; GE Healthcare, Slough, UK) was incubated with 10 ml of blocking buffer containing 5% (wt/vol) skimmed milk powder (Sigma-Aldrich, UK) in PBST (PBS with 0.05% Tween 20 [Sigma-Aldrich, UK]) overnight at 4°C. The membrane was then incubated for 2 h with a 1:1,000 dilution of pooled bovine convalescent-phase serum in PBST with 1% (wt/vol) skimmed milk and washed three times with PBST. Secondary antibody (goat anti-bovine IgG-HRP; Santa Cruz Biotechnology, Heidelberg, Germany) was added at a concentration of 400 ng/ml for 1 h at room temperature. The blot was washed again. Immunoreactivity was visualized by chemiluminescence from ECL.

### T cell proliferation assays.

Blood was obtained from Holstein-Friesian cattle aged 18 to 36 months via jugular vein puncture. Animals were reared indoors and maintained on a ration of hay and concentrates. PBMCs were isolated by density gradient centrifugation using Ficoll Paque plus (GE Healthcare, UK) as described previously (45). Human PBMCs were isolated from venous blood drawn from healthy human volunteers and mixed with acid-citrate-dextran (ACD) (25 g d-glucose [Sigma-Aldrich, UK] and 20.5 g trisodium citrate [Sigma-Aldrich, UK] added to 1 liter of double-distilled water [ddH_2_O]). The buffy coat was isolated by spinning the blood at 1,500 × *g* for 15 min with no break, and PBMCs were then isolated by using Ficoll Paque plus (GE Healthcare, UK) according to the manufacturer's specifications. PBMCs were adjusted to a concentration of 1 × 10^6^ cells/ml in complete cell culture medium (RPMI 1640 [Sigma-Aldrich, UK] supplemented with 10% [vol/vol] heat-inactivated fetal calf serum [Gibco, UK] and 100 U/ml penicillin, 100 μg/ml streptomycin, and 292 μg/ml l-glutamine. [PSG] [Gibco, UK]) and stimulated at least in triplicate with the concentrated total protein S. aureus supernatant fraction or recombinant protein. Culture medium and 50 μg/ml concanavalin A were used as negative and positive controls, respectively. Proliferations of bovine and human PBMCs were assessed by a [^3^H]thymidine incorporation assay as described previously (14). Total RNA was extracted from bovine PBMCs (4 × 10^6^ cells) by using Tri reagent (Sigma-Aldrich, Dorset, UK) according to the supplier's instructions or by using the RNeasy plus kit (Qiagen, UK) according to the manufacturer's instructions. First-strand cDNA was generated from 0.5 μg of RNA using a Power SYBR green RNA-to-CT 2-step kit or a high-capacity RNA-to-cDNA kit and Power SYBR green PCR master mix (Thermo Fisher, UK). The reverse transcription reaction was performed with a 20-μl volume according to the manufacturer's specifications. Bovine Vβ subfamily-specific qRT-PCRs were carried out as described previously (14). Human Vβ activation analysis was performed as described previously (12, 46).

### Experimental infection of dairy cattle.

Adult cows (Holstein) in their 1st to 4th lactation at 92 to 174 days in milk (DIM) were used in this study. Cultures of S. aureus grown overnight were inoculated 1:50 into fresh TSB and grown until an OD_600_ of 1.1 was reached. Staphylococci were diluted in TSB to obtain an inoculum of 5 × 10^7^ CFU/ml. Inocula were determined by CFU enumeration following serial dilution, plating on TSA, and growth at 37°C. Animals were challenged via teat dip immersion twice daily (22-mm immersion) until a score of 1 or higher for milk appearance or udder evaluation was observed and the animal developed an intramammary infection twice within a 5-day period. Following infection, animals were observed for a total of 3 weeks. Somatic cell counts (SCCs) and cultures were performed twice a week. Udder and milk clinical scores and milk yield and milk conductivity data were collected at each milking, which was performed twice daily.

### Statistical analysis.

All statistical analysis was performed in GraphPad Prism 7. Fold change enrichment data were analyzed by using the Student *t* test with Welch's correction if required. Tests were unpaired and two tailed, and significant differences were considered when the *P* value was <0.05.

## Supplementary Material

Supplemental file 1
