# Supplementary material for: Bovine Staphylococcus aureus Superantigens Stimulate the Entire T Cell Repertoire of Cattle
Source: Infect Immun. 2018 Oct 25;86(11):e00505-18. doi: 10.1128/IAI.00505-18 (PMC6204692; doi:10.1128/IAI.00505-18)
Supplement: Supplemental file 1 [file zii999092591s1.pdf]

Supplementary Table 1: SAg reference protein sequences used and diversity amongst bovine isolates.

Superantigen	Reference (Accession number, NCBI)	Diversity of homologs in bovine isolates (relative to reference sequence)	Crossmatches (>60%)
SEA	WP_000750406	98-100	selp (84%), sej (73%)
SEB	WP_000278085	99-100	sec (75%), selu/selw (60%)
SEC	AAG29599	99-100	seb (75%), selu/selw (60%)
SED	WP_000714364	98-99	selp (60%),
SEE	WP_000750405	---	sea (84%), sej (71%)
SEG	WP_000736712	97-100	ser (59%)
SEH	WP_000608674	99-100	-
SEI	WP_000713847	96-100	sei (66%), sell (60%)
SEJ	WP_031875254	99-99	sej (60%), selp (62%)
SEK	WP_000733771	96-100	sei (66%), sell (60%)
SElL	WP_000746599	97-100	sei (60%), selk (60%), sei (60%)
SElM	WP_000821658	91-100	selv (83%), seq (65%)
SElN	WP_001236362	94-100	-
SElO	WP_000935742	91-100	-
SElP	YP_009113107	99-99	sea (84%)
SEQ	WP_001033320	98-100	selm (64%)
SER	BAC97795	99-99	seg (62%)
SES	WP_032492129	63-67	seln (41%)
SET	WP_031888057	---	-
SElV	WP_043859283	96-99	sec (61%), seb (64%)
SElU/U2	WP_000764692	93	selm (88%)
SElW	WP_000889280	94-100	-
SElX	WP_000475326	93-100	-
SElY	WP_000644941	99-100	-
SElZ	WP_001225809	99-100	selu/selw (38%), sec (34%)
TSST-1	WP_001035599	98-100	-

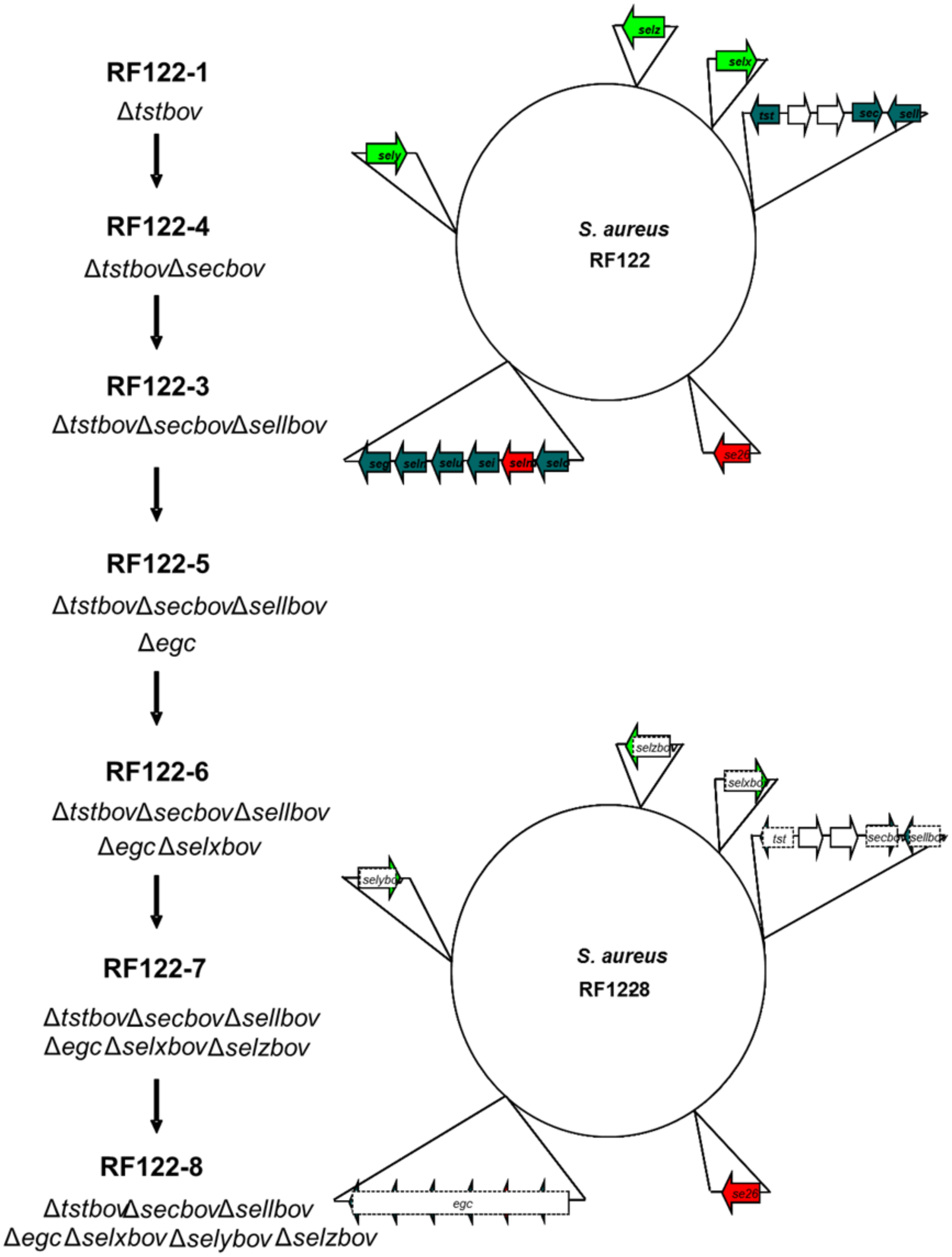

Figure S1: Sequential allele replacement events in *S. aureus* RF122-1 resulting in the generation of RF122-8. Schematic representation of the sequential deletion strategy used to prepare SAg deficient mutant RF122-8**.** Dark green arrows indicate bovine variants of characterised SAg genes, light green arrows denote putative SAg genes, and red arrows represent SAg pseudogenes. Unfilled arrows indicate genes encoding pathogenicity island proteins. Boxes with broken lines indicate deleted regions.

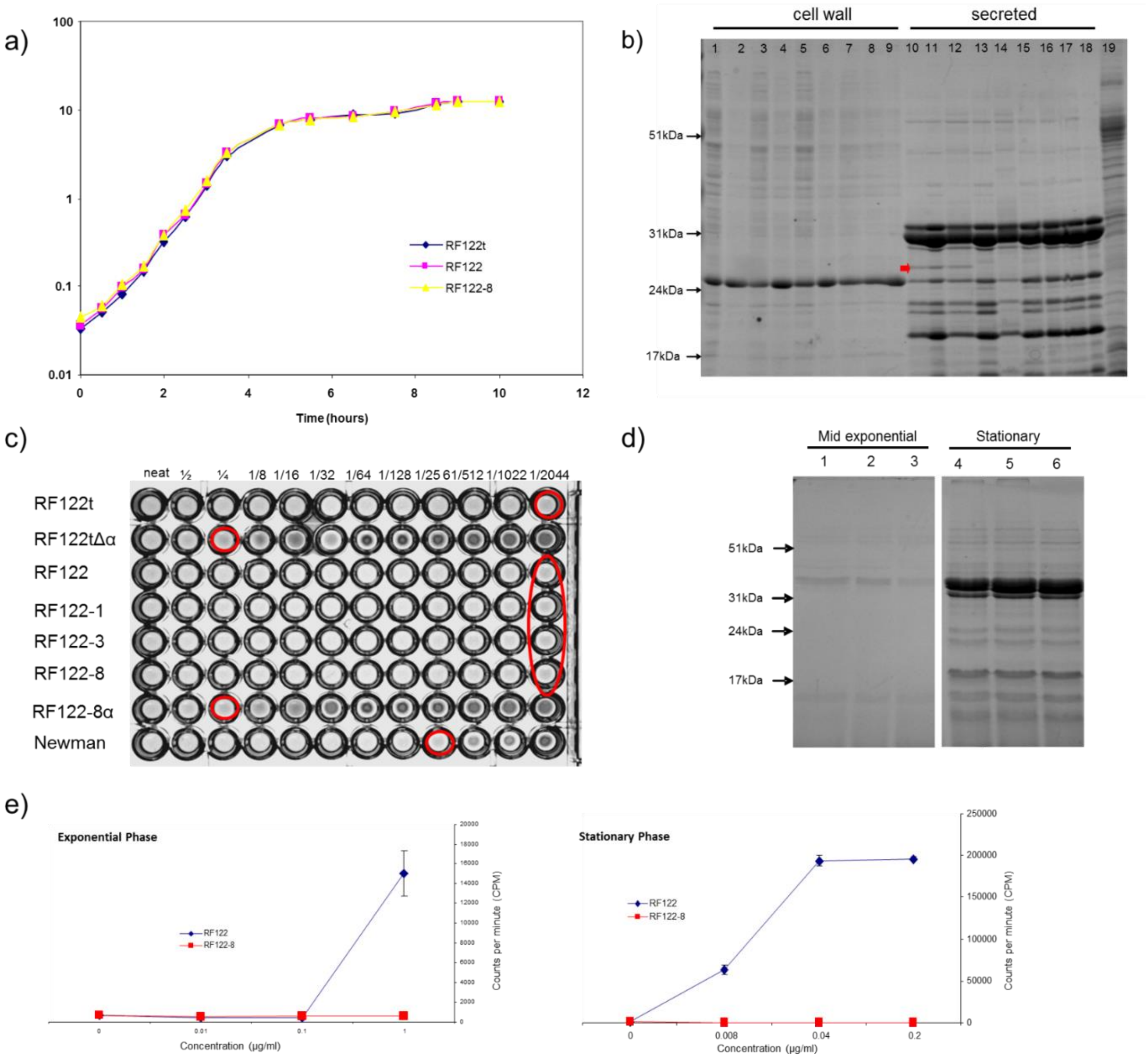

a)
Time (hours)
RF122t
RF122
RF122-8
b)
cell wall
secreted
51kDa
31kDa
24kDa
17kDa
c)
neat ½ ¼ 1/8 1/16 1/32 1/64 1/128 1/256 1/512 1/1022 1/2044
RF122t
RF122tΔα
RF122
RF122-1
RF122-3
RF122-8
RF122-8α
Newman
d)
Mid exponential
Stationary
51kDa
31kDa
24kDa
17kDa
e)
Exponential Phase
RF122
RF122-8
Counts per minute (CPM)
Concentration (µg/ml)
Stationary Phase
RF122
RF122-8
Counts per minute (CPM)
Concentration (µg/ml)

Figure S2: Phenotypic analysis of the RF122 and SAg-deficient derivative RF122-8 a) Growth curve of S. aureus strains RF122, RF122t and RF122-8 grown in BHI at 37 °C. b) Haemolytic activity of S. aureus strains Newman, RF122 and mutant strains. Experiments were performed at least in triplicate. End points of haemolysis have been circled in red. c) SDS-PAGE analysis of secreted and CWA proteins extracted from stationary phase cultures of S. aureus. SECbov protein is indicated by a red arrow. Lane 1, RF122; lane 2, RF122t; lane 3, RF122-1; lane 4, RF122-3; lane 5, RF122-4; lane 6, RF122-5; lane 7, RF122-6; lane 8, RF122-7; lane 9, RF122-8; lane 10, RF122; lane 11, RF122t; lane 12, RF122-1; lane 13, RF122-3; lane 14, RF122-4; lane 15, RF122- 5; lane16, RF122-6; lane 17, RF122-7; lane 18, RF122-8; lane 19, RN4220. d) Comassie stained SDS-PAGE analysis of mid-exponential and stationary phase protein supernates. Lanes 1 to 4 contain secreted proteins from exponential phase cultures of RF122t, and RF122-8, Lanes 5 to 8, secreted proteins from stationary phase cultures of RF122t and RF122-8. e) PBMC proliferation after 4 d exposure to S. aureus supernatants, as indicated by the incorporation of [3H] thymidine. Experiments were performed in triplicate from 2 cows and error bars were included to represent S.E.M.

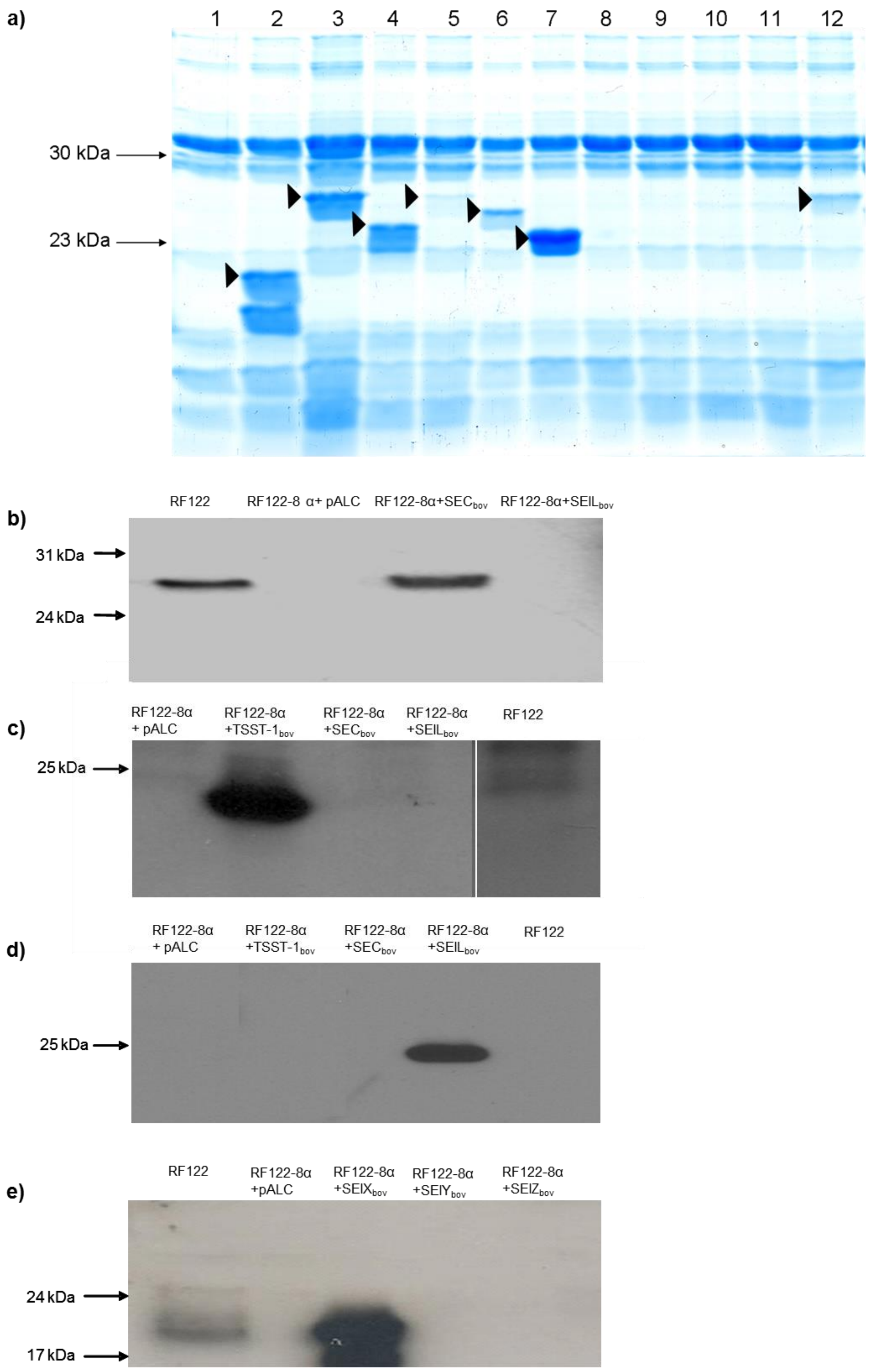

a)
1
2
3
4
5
6
7
8
9
10
11
12
30 kDa
23 kDa
b)
RF122
RF122-8 α+ pALC
RF122-8α+SEC$_{bov}$
RF122-8α+SEIL$_{bov}$
31 kDa
24 kDa
c)
RF122-8α + pALC
RF122-8α +TSST-1$_{bov}$
RF122-8α +SEC$_{bov}$
RF122-8α +SEIL$_{bov}$
RF122
25 kDa
d)
RF122-8α + pALC
RF122-8α +TSST-1$_{bov}$
RF122-8α +SEC$_{bov}$
RF122-8α +SEIL$_{bov}$
RF122
25 kDa
e)
RF122
RF122-8α +pALC
RF122-8α +SEIX$_{bov}$
RF122-8α +SEIY$_{bov}$
RF122-8α +SEIZ$_{bov}$
24 kDa
17 kDa

Figure S3: Expression of SAg genes in RF122-8α. RF122-8α supernatants expressing individual RF122-encoded SAgs, concentrated approx. 100-fold and resolved on SDS-PAGE gels and stained with Coomassie Blue. Lane 1, RF122-8α containing pALC2073; lane 2, RF122-8-X; lane 3, RF122-8-Z; lane 4, RF122-8-Y; lane 5, RF122-8-C, lane 6, RF122-8-L; lane 7, RF122-8-T; lane 8, RF122-8-G; lane 9, RF122-8-I; lane 10, RF122-8-O; lane 11, RF122-8-N; lane 12, RF122-8-U. Black arrows indicate where SAg proteins of the predicted molecular weight were expressed. **b)** Western blot analysis of concentrated RF122-8α supernatants containing SAgs separated by SDS-PAGE, with antibody specific for SEC_{bov}, **c)** TSST, **d)** SElL or **e)** $SElX_{bov}$.

(a)

```
RF122          KAKLWFLLTT LAFLIAVTES IGIAEVKAKT TGLITENSND SLKEHYAQKF EVYTNKEVTG VGENYIDTKV DTYNVRTVLY NTDYLKQFKN QDKVNIWGTL YENQQSKVYR GTVVKYDPIS [120]
ST151_Cattle   .......... .......... .......... .......... .......... .......... .......... .......... .......... .......... .......... .......... [120]
ST3140_Cattle  .......... .......... .......... .......... .......... .......... .......... .......... .......... .......... .......... .......... [120]
ST504_Cattle   .......... .......... .......... .......... .......... .......... .......... .......... .......... .......... .......... .......... [120]
ST705_Cattle   .......... .......... .......... .......... .......... .......... .......... .......... .......... .......... .......... .......... [120]
ST3099_Cattle  .......... .......... .......... .......... .......... .......... .......... .......... .......... .......... .......... .......... [120]
ST1569_Cattle  .......... ........G. .......... .......... .......... .......... .......V.. .......... .......... .......... .......... .......... [120]
ST9_Cattle     .......... ........G. .......... .......... .......... .......... .......V.. .......... .......... .......... .......... .......... [120]
ST50_Cattle    .......... ........G. .......... .......... .......... .......... .......... .......... .......... .......... .......... .......... [120]
ST20_Cattle    .......... ........G. .......... .......... .......... .......I.. .......A.. .......... .......... .......... ...K...... .......... [120]
MSA2020        .......... ........G. .......... .......... .......... .......... .......A.. .......... .......... .......... .......... .......... [120]
ST121_Human    .......... ........G. .......... .......... .......... .......... .......A.. .......... .......... .......... .......... .......... [120]
ST20_Human     .......... ........G. .......... .......... .......... .......I.. .......A.. .......... .......... .......... ...K...... .......... [120]
ST338_Human    .......... ........G. .......... .......... .......... .......... .......... .......... .......... .......... .......... .......... [120]
ST27_Human     .......... ........G. .......... .......... .......... .......... .......V.. .......... .......... .......... .......... .......... [120]
ST80_Human     .......... ........G. .......... .......... .......... .......... .......A.. .......... .......... .......... .......... .......... [120]
ST59_Human     .......... ........G. .......... .......... .......... .......... .......... .......... .......... .......... .......... .......... [120]
ST292_Human    .......... ........G. .......... .......... .......... .......... .......... .......... .......... .......... .......... .......... [120]

RF122          KVTNLSYRMN LFVNGHQTKV NPDSLLEVKN KQISLKETDF RIRKYLLEKE HLYSNYNSGE LIIEMKNGAR HKIDLGDILS DSQEKTFDFD NISHIDIYMK [220]
ST151_Cattle   .......... .......... .......... .......... .......... .......... .......... .......... .......... .......... [220]
ST3140_Cattle  .......... .......... .......... .......... .......... .......... .......... .......... .......... .......... [220]
ST504_Cattle   .......... .......... .......... .......... .......... .......... .......... .......... .......... .......... [220]
ST705_Cattle   .......... .......... .......... .......... .......... .......... .......... .......... .......... .......... [220]
ST3099_Cattle  .......... .......... .......... .......... .......... .......... .......... .......... .......... .......... [220]
ST1569_Cattle  .......... .......... .......... .......... .......... .......... .......... .......... .......... .......... [220]
ST9_Cattle     .......... .......... .......... .......... .......... .......... .......... .......... .......... .......... [220]
ST50_Cattle    .......... .......... .......... .......... .......... .......... .......... .......... .......... .......... [220]
ST20_Cattle    .......... .......... .......... .......... .......... .......... .......... .......... .......... .......... [220]
MSA2020        .......... .......... .......... .......... .......... .......... ..V....... .......... .......... .......... [220]
ST121_Human    .......... .......... .......... .......... .......... .......... ..V....... .......... .......... .......... [220]
ST20_Human     .......... .......... .......... .......... .......... .......... .......... .......... .......... .......... [220]
ST338_Human    .......... .......... .......... .......... .......... .......... .......... .......... .......... .......... [220]
ST27_Human     .......... .......... .......... .......... .......... .......... .......... .......... .......... .......... [220]
ST80_Human     .......... .......... .......... .......... .......... .......... .......... .......... .......... .......... [220]
ST59_Human     .......... .......... .......... .......... .......... .......... .......... .......... .......... .......... [220]
ST292_Human    .......... .......... .......... .......... .......... .......... .......... .......... .......... .......... [220]
```

(b)

```
RF122           ETQNDPNISE LNKSSQYTGS WHNIWYLYNS DPVNAKKIKL SDKFLSHDFI VPINNPGHYD YVKTELKDST MASSFDGKEV DIFGVNYFDQ CYFSNENIQC DSNQGGSKK TCMYGGITLN  [120]
ST151_Cattle    .......... .......... .......... .......... .......... .......... .......... .......... .......... .......... .......... ..........  [120]
ST705_Cattle    .......... .......... .......... .......... .......... .......... .......... .......... .......... .......... .......... ..........  [120]
ST3140_Cattle   .......... .......... .......... .......... .......... .......... .......... .......... .......... .......... .......... ..........  [120]
ST504_Cattle    .......... .......... .......... .......... .......... .......... .......... .......... .......... .......... .......... ..........  [120]
ST3099_Cattle   .......... .......... .......... .......... .......... .......... .......... .......... .......... .......... .......... ..........  [120]
ST12_Cattle     .......... .......... .......... .......... .......... .......... .......... .......... .......... .......... .....A.... ..........  [120]
ST71_Cattle     .......... .......... .......... .......... .......... .......... .......... .......... .......... .......... .......... ..........  [120]
MSA1690         .....L.... .......... .......... .......... .......... ....S..... .......... .....N.... .......... .......... .....A.... ..........  [120]
ST45_Human      .......... .......... .......... .......... .......... ....S..... .......... .......... .......... .......... .....A.... ..........  [120]
ST12_Human      .......... .......... .......... .......... .......... .......... .......... .......... .......... .......... .....A.... ..........  [120]
ST121_human     .......... .......... .......... .......... .......... .......... .......... .......... .......... .......... .......... ..........  [120]
ST123_Human     .......... .......... .......... .......... .......... .......... .......... .......... .......... .......... .......... ..........  [120]
ST772_Human     .......... .......... .......... .......... .......... .......... .......... .......... .......... .......... .....A.... ..........  [120]
ST446_Human     .......... ...A...... .......... .......... .......... ....S..... .......... .......... ........H. .......... .....A.... ..........  [120]
ST93_Human      .......... ...A...... .......... ...K...T.. .......... ....S..... .......... .......... ........H. .......... .....A.... ..........  [120]

RF122           ENNTNNRIQP IVVKVYENDS VTLSFDINID KETVTIQELD YKVRNKLISK INLYHLGGTS YETGYIKFIE NGNRYYWYDM MPDPGFTQSK YLMIYRGNET VESAKTEIEV HLTKK [235]
ST151_Cattle    .......... .......... .......... .......... .......... .......... .......... .......... .......... .......... .......... ..... [235]
ST705_Cattle    .......... .......... .......... .......... .......... .......... .......... .......... .......... .......... .......... ..... [235]
ST3140_Cattle   .......... .......... .......... .......... .......... .......... .......... .......... .......... .......... .......... ..... [235]
ST504_Cattle    .......... .......... .......... .......... .......... .......... .......... .......... .......... .......... .......... ..... [235]
ST3099_Cattle   .......... .......... .......... .......... .......... .......... .......... .......... .......... .......... .......... ..... [235]
ST12_Cattle     .......... .......... .......... .......... .......... .......... .......... .......... .......... .......... .......... ..... [235]
ST71_Cattle     .......... .......... .......... .......... .......... .......... .......... .......... .......... .......... .......... ..... [235]
MSA1690         .......... .......... .......... .......... .......... .......... .......... .......... .......... .......... .......... ..... [235]
ST45_Human      .......... .......... .......... .......... .......... .......... .......... .......... .......... .......... .......... ..... [235]
ST12_Human      .......... .......... .......... .......... .......... .......... .......... .......... .......... .......... .......... ..... [235]
ST121_human     .......... .......... .......... .......... .......... .......... .......... .......... .......... .......... .......... ..... [235]
ST123_Human     .......... .......... .......... .......... .......... .......... .......... .......... .......... .......... .......... ..... [235]
ST772_Human     .......... .......... .......... .......... .......... .......... .......... .......... .......... .......... .......... ..... [235]
ST446_Human     .......... .......... .......... .......... .......... .......... .......... .A........ ....S..... .......... .......... ..... [235]
ST93_Human      .......... .......... .......... .......... .......... .......... .......... .A........ ....S..... .......... .......... ..... [235]
```

Figure S4: Amino acid alignment of allelic variants of SElY and SElZ identified in the whole genome dataset. Amino acid sequences of SElY**(a)** and SElZ **(b)** were identified by the distribution analysis of *S. aureus* genomes. A representative of each variant from each lineage, for both cattle or human isolates is shown and aligned. Dots signify conservation with the corresponding location in SElY or SElZ from RF122. Residues that differ between the allele from RF122 and the allele used as the human variant in phenotypic studies is highlighted in green.

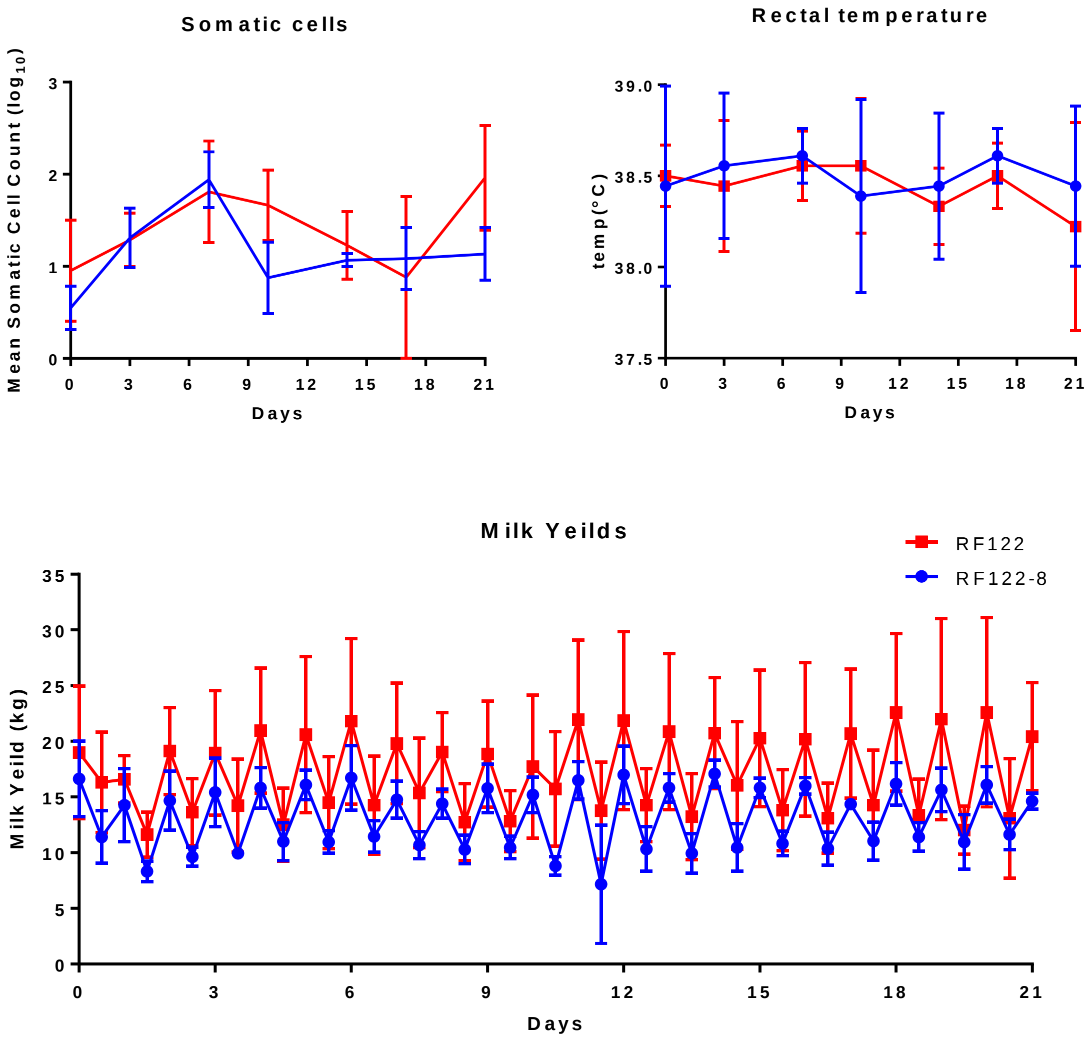

Figure S5: SAgs do not affect the presentation of sub-clinical mastitis. Measures that indicate sub-clinical mastitis were taken throughout the bovine infection study. Mean data from each treatment group ± SD for somatic cells, rectal temperatures and milk yields.

Supplementary Table 2: Primers used in this study.

Primer	Forward sequence (5´-3´) [a, b]	Reverse sequence (5´-3´) [a]
sely	TGGTTACAGTAGCTATTCTTTGTTGG	TTAGTTAAGTGCACTTCTATTTCCGTT
selz	AGCGAAACTATGGTTTTTATTGAC	CTTGTGAGTCACTCAATATGTCGC
16srRNA	TATGGAGGAACACCAGTGGCGAAG	TCATCGTTTACGGCGTGGACTACC
*selx*q	AGCAGACGCGTCAACACACAAA	GGTCTCTCTGAATAAACCCAATTCC
*sely*q	GTTGGAAGCTAGAGCAAGACA	GCCAAGAACCCGTATTGACT
*selz*q	CCTACAATGTACGGACAGTGCTCTAC	GGATCATACTTAACTACTGTGCCACG
*tst*q	TGGTATAGTAGTGGGTCTGACGCT	AGGCTGATGTGCCATCTGTGTTT
*sec*q	AACAGTTCACCATATGAAACAGGT	AGATTGGTCAAACTTATCTCCTGGT
*sell*q	ATCTAACGGCGCGATGTAGGTCCA	CTAAGCGGTGTGATTCTGGTGA
*seg*q	TGCGAATGCCCTACCTGATCCTAA	TCCTCTTCCTTCAACAGGTGGAGA
*seln*q	CATCGTGCTTAAACGGAGAGT	ACCTTCTTGTTGGACACACCATCT
*selu*q	AGTTCGCCTTATGAGACTGGCT	TCACCAGATTTCAGATTCAGGCATCA
*sei*q	GCGCAAGGAGATTGGTGTAGGT	GATCAAATCATTGGGACCGGTTG
*selm*q	TCGTCTGTTGATGTATACGGCCT	ACCCGCTAAAGTAACTCCTCCGTA
*selo*q	TTGTCATGGTGAGCATCAAGTGAAA	GCTACTCCTATTTCTTTAGGTTCGT
*se26*q	AACGCAATGTAGTTATGGTGGTGT	TCTGTTTGATGTCCGTCAATCCAT
*selx*pET	TAGCCATATGTCAACACAAAATTCCTCAA	GCGCGGATCCTCAAACTTGTTCAATGTC
*sely*pET	GCGCCATATGAAAACAACTGGATTGATTA	GCGCGGATCCCTATTTCATATAAATATC
*selz*pQE30	TAGCGGATCCGAGACACAAAATGATCCAAA	GCGCCTGCAGCTACTTTTTAGTTAAGT
*seg*pET	GCGCCATATGCTACCTGATCCTAA	GCGCGGATCCTTATATATTCAGATTC

*sei*pET	GCGCCTCGAGGATATTGGTGTAGGTA	GCGCCTCGAGTTAGTTACTATCTACATA
*selo*pET	GCGCCATATGAATGAAGAAAATCCTA	GCGCGGATCCTTATGTAAATAAATAAAC
*selu*pET	GCGCCATATGAATAAAGCGAGTGAAT	GCGCGGATCCCTTATTTTTTGGTTAA
T7	AAATTAATACGACTCACTATAGG	GCTAGTTATTGCTCAGCGGT
*secbov*AB	ATGAATTCCTGTGGATTTAGAAATAAGG	CCAACATTCCCAAGAAGTATC
*secbov*CD	**GATACTTCTTCTTGGGAATGTTGG**AAGAATGGATAATGTTAATCC	TTATCCATGGCAAGCATCAAAC
*secbov*EZ	GTCATGTTTCGGTTGATAGG	ATGGCGGTGTTACTAAAGC
up*secbov*	GCAGGTACTTCGGTACTTGCCTAT	GGAGAAACAGAGGATTTCTAAGCATC
*Secbov*probe	TGAAGGAAACCACTTTGATAATGGG	AGATTGGTCAAACTTATCTCCTGGT
*sell*AB	GATATATTTGAAAGGTAAGTACTTCG	AGTGTAGTATTCCATATGAATGATGGT
*sell*CD	**ACCATCATTCATATGGAATACTACACT**ATACAAAAGGTTATAGGAAGAGTT	CAATTTCTACAGATATGACTCCC
*sell*EZ	GTCATGTTTCGGTTGATAGG	TGTACAAATGGACTTAAGATATAGCG
*egc*AB	TCTTGATACGTATTTGACACTTGC	AGCTATACGAGTTTGATGGTTCTG
*egc*CD	**CAGAACCATCAAACTCGTATAGCT**AACTAAGCGACTCAGATAATAGAC	AGAGTTGTTACAGTCGCTACACC
*egc*EZ	AGCACAAACTGTAGCAGAACATGAG	GTATATAGTAGGAATGAACTATATAGCC
up*egc*	GAAGAAGTATTTGAATTCTTATATGA	CATCATTTACAATTATTAACATGAT

	CC	AGG
*selx*AB	TGTCGATGCTATGGATAGTGAGG	TAATTACCTCCTTGATGTAAAGC
*selx*CD	**GCTTTACATCAAGGAGGTAATTAT**ATCGCTAATACTTTGAAAGTTAGG	TCAAATGTAGCAGTATACATTAATTGCG
*selx*EZ	ATCTCAGTTGTCTCTTTGATAGTGC	TTACTCTTCATGTGTCACTTCATTTCG
*selx*probe	AATCTAATAAAGCTAGGAATTGGG	TGTAACTCTTTGTGCGACTCTAATG
up*selx*	GTGTATCTTAATTTCATATCTATAGTTGC	AAGCAATGCAAGAGCATGTAGG
*sely*AB	ACTTGAAGGTATGTATGTTACACG	TCTAGCTTCCAACAAAGAATAGC
*sely*CD	**GCTATTCTTTGTTGGAAGCTAGAG**GTAATGAAACAGTTGAATCAGC	AAGAACCACGCGAACACACAAGC
*sely*EZ	AGATTATTAGGTAAGGCATAAATAGC	TCAGCTTACAATAATGTGCAAGTTGG
*sely*probe	GAAAGAGTAACACTGTCATTCTCG	TTGAGTCATGACTTCATTGTTCC
up*sely*	ATTCTAATGTTTACGTTCTTCATAGC	CACAAATTGCAATTACTGTTATACG
*selz*AB	AAGGTGACAATCCTGAAATCAC	CAGATCATCCTTTCTCATTTAAGATT
*selz*CD	**AATCTTAAATGAGAAAGGATG**ATCTGGTTCCAACATAAATGCG	TTATGGCTCAGGTTCAGTTGGT
*selz*EZ	TAGGACGATTGGAACTTGTGAG	ATATGAAGATGCTGTAGATTATAACC
*hla*AB	ATTCATCATTAGAAGCTAACCTATACTC	GATTTGAGGAAACAATAATCAATATGTC
*hla*CD	**GACATATTGATTATTGTTTCCTCAAATC**TGTAAATTGTTTGTTCAT	TCAGAACCATAGTTGACATGAGC
*hla*EZ	GAAATTTTATAGCCTGATTCAGACTC	TTATCTAATTTTCATTTGCTTTACATG

pMADMCS	AAGCGAGAATCATAATGGG	CTTGCTCCAACTGAAAATCCC
*sec*pALC	TCTAGGTACCTCTTGGGAATGTTGG	TATCGAGCTCGGATTAACATTATCC
*sell*pALC	TTATGGTACCTATAATGAAAAGGAAGTGC	AATCTGAGCTCTAATAATTGGAATCATC
*tst*pALC	AAACGGTACCACATTTAAATGAAGG	AAAAGAGCTCAATTAATTAATTTCTGC
*seg*pALC	CATTGGTACCTAGACTGAATAAGTTAGAGG	ATTTGAGCTCTTCAGTAAATTTTATATATTC
*sei*pALC	CAATGGTACCTTAGAAAAGGAAATGC	TTTAGAGCTCAAACTAATTATCATTAG
*selo*pALC	TTTTGGGTACCAGGGATATTATAA	TTGTGAGCTCATATGCATCAACTT
*seln*pALC	TTGAGGTACCGTATATTATAAA	GATTGAGCTCATAATCATCAATCACTT
*selu*pALC	TACCTGGTACCCATCAAATTTATAAA	TAATGAGCTCTCCCTCAATTA
*selx*pALC	TAATGGTACCAAATAAAGCTTTACATC	AATTGAGCTCAATTTCAAACTTGTTC
*sely*pALC	CAATGGTACCTGAAGGTCAACTAA	TAAGGAGCTCTATGTCTACTTTTTAGTT
*selz*pALC	TTTGGTACCTTTAATCTTAAATGAGA	GGAACGAGCTCTTTCATATAAATATCT
MCSpALC	ATACCGCACAGATGCGTAAGG	CGATGACTTAGTAAAGCACATCTAA

[a] Restriction sites incorporated are underlined.

[b] Complementary regions of allele replacement primers are in bold type.

Supplementary Table 3: Bacterial strains and plasmids used in this study

Strain or plasmid	Description	Source/Reference
**Plasmids**		
pSC-B	Contains *lacZ*' α-complementation cassette	Stratagene
pMAD	Thermosensitive gene replacement plasmid, pE194 derivative	(1)
pMAD:*secbov*	*secbov* deletion construct	This study
pMAD:*sellbov*	*sellbov* deletion construct	This study
pMAD:*egc*	*egc* deletion construct	This study
pMAD:*selxbov*	*selxbov* deletion construct	This study
pMAD:*selybov*	*selybov* deletion construct	This study
pMAD:*selzbov*	*selzbov* deletion construct	This study
pMAD:*hla*	*hla* deletion construct	This study
pALC2073	Expression plasmid; pSK236 containing the *tetR* gene and the *xyl/tetO* promoter	(2)
pALC2073::SEC_{bov}	*secbov* cloned into pALC2073	This study
pALC2073::$SElL_{bov}$	*sellbov* cloned into pALC2073	This study
pALC2073::$TSST\text{-}1_{bov}$	*tstbov* cloned into pALC2073	This study
pALC2073::SEG_{bov}	*segbov* cloned into pALC2073	This study
pALC2073::SEI_{bov}	*seibov* cloned into pALC2073	This study
pALC2073::$SElO_{bov}$	*selobov* cloned into pALC2073	(3)
pALC2073::$SElN_{bov}$	*selnbov* cloned into pALC2073	This study
pALC2073::$SElU_{bov}$	*selubov* cloned into pALC2073	This study
pALC2073::$SElY_{bov}$	*selybov* cloned into pALC2073	This study
pALC2073::$SElZ_{bov}$	*selzbov* cloned into pALC2073	This study
pALC2073::$SElX_{bov}$	*selxbov* cloned into pALC2073	This study
pET15b	Cloning vector, carries an N-terminal His	Novagen

	Tag sequence	
pQE30-xa	Cloning vector, carries an N-terminal His Tag sequence	Qiagen
pET::SElX$_{bov}$	*selxbov* cloned into *Nde*1 and *Bam*HI sites of pET15b	(3)
pET::SElY$_{bov}$	*selybov* cloned into *Nde*1 and *Bam*HI sites of pET15b	This study
pET::SElY$_{hu}$	*selyhu* cloned into *Nde*1 and *Bam*HI sites of pET15b	This Study
pQE30-Xa::SElZ$_{bov}$	*selzbov* cloned into *Bam*HI and *Pst*I sites of pQE30-Xa	This Study
pQE30-Xa::SElZ$_{hu}$	*selzhu* cloned into *Bam*HI and *Pst*I sites of pQE30-Xa	This Study
pET::SEG	*segbov* cloned into *Nde*1 and *Bam*HI sites of pET15b	This study
pET::SEI	*seibov* cloned into *XhoI* site of pET15b	This study
pET::SElO	*selobov* cloned into *Nde*1 and *Bam*HI sites of pET15b	This study
pET::SElU	*selubov* cloned into *Nde*1 and *Bam*HI sites of pET15b	This study
E. coli		
DH5α	Cloning strain	Invitrogen
BL21(DE3)	Expression Strain	Invitrogen
XL-1 Blue	Expression Strain	Agilent
Strataclone SoloPack™	*lacZΔM15* mutation, *endA, recA* deficient	Stratagene
S. aureus		
RF122	Wild type bovine mastitis	(4)

RF122t	Transducable variant of RF122	(5)
RF122-1	*tst*::Tc^r	(5)
RF122-3	*tst*::Tc^r Δ*sec*	This study
RF122-4	*tst*::Tc^r Δ*sec*Δ*sell*	This study
RF122-5	*tst*::Tc^r Δ*sec*Δ*sell*Δ*egc*	This study
RF122-6	*tst*::Tc^{r}Δ*sec*Δ*sell*Δ*egc*Δ*selx*	This study
RF122-7	*tst*::Tc^{r}Δ*sec*Δ*sell*Δ*egc*Δ*selx*Δ*selz*	This study
RF122-8	*tst*::Tc^{r}Δ*sec*Δ*sell*Δ*egc*Δ*selx*Δ*sely* Δ*selz*	This study
RF122t Δα	Δ*hla*	This study
RF122-8α	Δ*hla*	This study
RN4220	Restriction/modification$^-$ derivative of NTCC8325	(6)
RF122-8α-C	RF122-8α containing pALC2073::SEC$_{bov}$	This study
RF122-8α-L	RF122-8α containing pALC2073::SElL$_{bov}$	This study
RF122-8α-T	RF122-8α containing pALC2073::TSST-1	This study
RF122-8α-G	RF122-8α containing pALC2073::SEG$_{bov}$	This study
RF122-8α-I	RF122-8α containing pALC2073::SEI$_{bov}$	This study
RF122-8α-U	RF122-8α containing pALC2073::SElU$_{bov}$	This study
RF122-8α-N	RF122-8α containing pALC2073::SElN$_{bov}$	This study
RF122-8α-O	RF122-8α containing pALC2073::SElO$_{bov}$	This study
RF122-8α-X	RF122-8α containing pALC2073::SElX$_{bov}$	This study
RF122-8α-Y	RF122-8α containing pALC2073::SElY$_{bov}$	This study
RF122-8α-Z	RF122-8α containing pALC2073::SElZ$_{bov}$	This study

References

1. **Arnaud M, Chastanet A, Debarbouille M.** 2004. New Vector for Efficient Allelic Replacement in Naturally Nontransformable, Low-GC-Content, Gram-Positive Bacteria. Appl Environ Microbiol **70:**6887-6891.
2. **Bateman BT, Donegan NP, Jarry TM, Palma M, Cheung AL.** 2001. Evaluation of a tetracycline-inducible promoter in Staphylococcus aureus in vitro and in vivo and its application in demonstrating the role of sigB in microcolony formation. Infect Immun **69:**7851-7857.
3. **Wilson GJ, Seo KS, Cartwright RA, Connelley T, Chuang-Smith ON, Merriman JA, Guinane CM, Park JY, Bohach GA, Schlievert PM, Morrison WI, Fitzgerald JR.** 2011. A Novel Core Genome-Encoded Superantigen Contributes to Lethality of Community-Associated MRSA Necrotizing Pneumonia. PLoS Pathog **7:**e1002271.
4. **JR Fitzgerald WM, PJ Hartigan , CJ Smyth , V Kapur** 1997. Fine-structure molecular epidemiological analysis of Staphylococcus aureus recovered from cows. Epidemiology and Infection **119:**261-269.
5. **Fitzgerald JR, Monday SR, Foster TJ, Bohach GA, Hartigan PJ, Meaney WJ, Smyth CJ.** 2001. Characterization of a putative pathogenicity island from bovine Staphylococcus aureus encoding multiple superantigens. J Bacteriol **183:**63-70.
6. **Kreiswirth BN, Lofdahl S, Betley MJ, O'Reilly M, Schlievert PM, Bergdoll MS, Novick RP.** 1983. The toxic shock syndrome exotoxin structural gene is not detectably transmitted by a prophage. Nature **305:**709-712.
